# A Power Spectral Density-Based Method to Detect Tremor and Tremor Intermittency in Movement Disorders

**DOI:** 10.3390/s19194301

**Published:** 2019-10-04

**Authors:** Frauke Luft, Sarvi Sharifi, Winfred Mugge, Alfred C. Schouten, Lo J. Bour, Anne-Fleur van Rootselaar, Peter H. Veltink, Tijtske Heida

**Affiliations:** 1 Department of Biomedical Signals and Systems, University of Twente, 7522 NB Enschede, The Netherlands; 2 Amsterdam Neuroscience, Amsterdam UMC, Department of Neurology, University of Amsterdam, 1105 AZ Amsterdam, The Netherlands; 3 Department of Mechanical, Maritime and Materials Engineering, Delft University of Technology, 2600 AA Delft, The Netherlands; 4 Department of Biomechanical Engineering, University of Twente, 7522 NB Enschede, The Netherland

**Keywords:** accelerometers, automatic detection, electromyography, movement disorders, essential tremor, Parkinson’s disease, tremor, tremor stability index

## Abstract

There is no objective gold standard to detect tremors. This concerns not only the choice of the algorithm and sensors, but methods are often designed to detect tremors in one specific group of patients during the performance of a specific task. Therefore, the aim of this study is twofold. First, an objective quantitative method to detect tremor windows (TWs) in accelerometer and electromyography recordings is introduced. Second, the tremor stability index (TSI) is determined to indicate the advantage of detecting TWs prior to analysis. Ten Parkinson’s disease (PD) patients, ten essential tremor (ET) patients, and ten healthy controls (HC) performed a resting, postural and movement task. Data was split into 3-s windows, and the power spectral density was calculated for each window. The relative power around the peak frequency with respect to the power in the tremor band was used to classify the windows as either tremor or non-tremor. The method yielded a specificity of 96.45%, sensitivity of 84.84%, and accuracy of 90.80% of tremor detection. During tremors, significant differences were found between groups in all three parameters. The results suggest that the introduced method could be used to determine under which conditions and to which extent undiagnosed patients exhibit tremors.

## 1. Introduction

Tremors are the most common symptom in movement disorders [1]. The most common types of tremors are physiological (8–12 Hz, low amplitude), essential (4–12 Hz) [2], and parkinsonian (3–8 Hz) [2]. In contrast to a physiological tremor, essential and parkinsonian tremors are often disabling and lower the quality of life of patients. In clinical practice, diagnosis is based on family history and examination by a neurologist or movement disorder specialist, leading to a misdiagnosis in 20–37% of the patients [3,4,5]. Even though parameters, such as amplitude and presence of tremor, can be measured objectively and accurately using accelerometers and electromyography (EMG) [2,6,7,8,9,10], there is no objective gold standard to detect tremor episodes [11]. This involves the choice of the algorithm, as well as the choice of sensors. Furthermore, tremor detection methods are often used to detect tremors in one specific group of patients during the performance of a specific task. However, even within the same patient group, and certainly between patient groups, tremors may appear at different frequencies, during different tasks, and with varying amplitudes [7,12].

Studies show that differentiation between pathological tremors often requires long-term (hours) recordings [9,10], which makes these methods inadequate to implement in a clinical setting where examination times are rather short. Ghassemi et al. [13] used combined accelerometer and EMG measurements to differentiate between an essential tremor (ET) and a Parkinson’s disease (PD) tremor, achieving an overall accuracy of differentiation of 83%. Recently, di Biase et al. [14] introduced the tremor stability index (TSI) as a new promising tool for differentiation between tremor disorders. They used 100 s of tremor recordings and determined the change in tremor frequency with respect to the instantaneous frequency. However, it is not mentioned how the presence of tremor in these recordings is determined. Furthermore, to acquire 100 s of tremor recordings, long recording times might be necessary in some patients due to the intermittency of tremor. Additionally, the use of a tremor frequency band between 2–9 Hz could make it difficult to use the method to detect kinetic tremors because ET patient can experience tremors of up to 12 Hz.

Heida et al., in 2013 [15], divided data into tremor windows (TWs) and non-tremor windows (NTWs) to determine the effect of deep brain stimulation on tremor during rest and movement in patients with PD. In that study, it was shown that, during a TW, almost all power is concentrated around the tremor frequency, and in an NTW, the power distribution is shifted to the movement (0.25–3.5 Hz) and physiological tremor (7.5–20 Hz) frequency bands [15].

Therefore, the aim of this study was twofold. Firstly, we developed a transparent quantitative method to detect TWs in tremor disorder patients with a straightforward technical setup using only two accelerometers placed on the back of the hands and EMG recordings of the extensor carpi radialis muscles from both forearms. For the tremor detection method, we adopted the following criteria:

Pathological tremors need to be detected and distinguished from physiological tremors.

1. A large tremor frequency range needs to be adopted [16,17]. A frequency range of 3.5–12 Hz covers most pathophysiological tremors [18], including the most common ones: parkinsonian, essential, and physiological tremors.
2. Pathological tremors need to be detectable during various tasks.

We demonstrated that detection of tremors in accelerometer and EMG data is possible using a single method. The advantage of using both types of recordings is that different information is provided by them. EMG is related to motor unit recruitment and may be used to detect synchronization between (antagonistic) muscles, while accelerometer data is related to movements, which is the effective output resulting from the simultaneous (co-productive and counterproductive) activity of several muscles. 

Secondly, the TSI was determined by the TW and NTW to indicate the importance of splitting data before further analysis was applied.

## 2. Materials and Methods

### 2.1. Subjects and Clinical Evaluation

Ten patients with ET (8 males), age 61.9 ± 13.0 years (mean ± standard deviation), ten PD patients (7 males), age 64.6 ± 12.4 years, and ten healthy controls (HC) (7 male), age 59.7 ± 6.5 years participated in this study. Table 1 provides patient details. All subjects were right-handed according to the Edinburgh Handedness Inventory [19], and all patients were off tremor-suppressing medication (tapered on an individual basis). HC had no history of neurological disorders nor tremor. Furthermore, PD patients were diagnosed according to the UK Brain Bank criteria for Parkinson’s disease [20] and showed neither major fluctuation in symptoms nor suffer from severe dyskinesia. ET patients were diagnosed according to the criteria defined by the Tremor Investigation Group [21] and had a positive family history of ET. All subjects gave written informed consent in accordance with the Declaration of Helsinki prior to participation, and the study was approved by the medical ethics committee of the Academic Medical Center in Amsterdam, The Netherlands.

### 2.2. Experimental Protocol and Data Acquisition

All subjects were seated comfortably on a bed, with the back elevated to an upright position. Voluntary and involuntary movements were recorded with two 3D accelerometers (ACC) (TMSi, Oldenzaal, The Netherlands), one placed on the back of each hand, approximately in the middle of the third metacarpal bone, with the x-direction pointing towards the fingertips. Sensor size was 13 × 10 × 5 mm and sensor weight was 2 grams. Muscle activity was recorded from the m. extensor carpi radialis of both arms using surface EMG. Data acquisition was done using a custom-made program written in LabVIEW (National Instruments, Austin, TX, United States) with a sampling frequency of 2048 Hz. Subjects were asked to perform three tasks to evoke either resting, postural, or kinetic tremors:

1. Resting task (RT): Subjects sit with both hands in supine position resting in their lap; task duration: one min.
2. Postural task (PT): Subjects stretch out both arms, unsupported against gravity, approximately parallel to the floor; task duration: one min.
3. Movement task (MT): Subjects perform an elbow flexion-extension task (index finger from nose to knee) with the right arm at a self-paced speed, and the left arm remains rested in the lap; task duration: one min.

### 2.3. Data Analysis

Data analysis was performed offline in MATLAB (MathWorks, Inc., Natick, MA, USA, R2013a). Prior to analysis the dominant tremor axis of the 3D accelerometers was determined using principle component analysis (*pca* routine in MATLAB R2013a). Accelerometer data was band-pass filtered (non-causal, zero-phase, 0.5–20 Hz, 2nd-order Butterworth filter). EMG data was filtered (non-causal, zero-phase, 20–400 Hz, 4th-order Butterworth), and the absolute value of the Hilbert transform was used as the envelope of the signal for further analysis. For the detection of TWs and NTWs, data was divided into 3-s windows with an overlap of 1.5 s.

**Figure 1:**
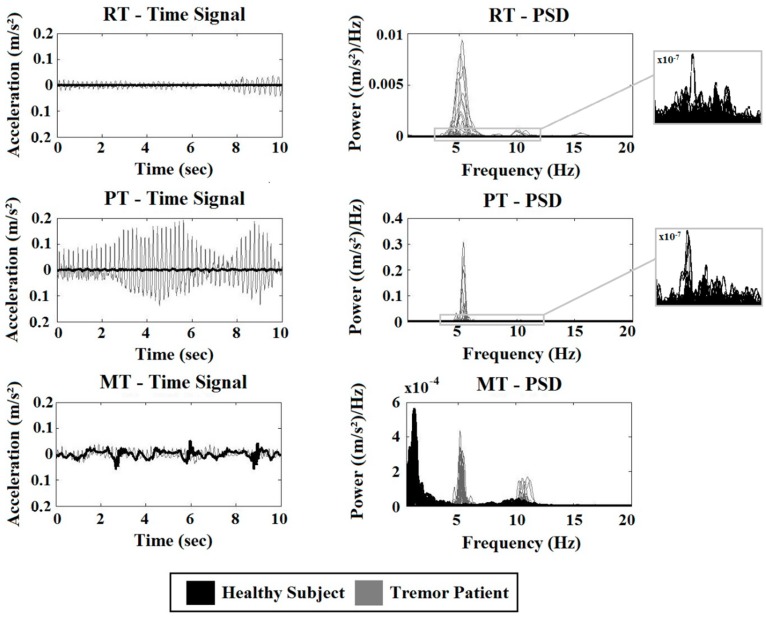
Time signals and corresponding power spectral densities (PSD) of a healthy control (**black**) and a tremor patient (**grey**). From top to bottom, data recorded during rest (RT), posture (PT), and movement (MT). On the right side, an enlargement of the PSD of the HC is given.

Prior to objective classification, each window (accelerometer and EMG) was classified as either TW or NTW by two raters (FL and TH), based on the power spectral density (PSD) of each 3-swindow, which was estimated using the periodogram function in MATLAB (see Figure 1A). Discrepant ratings were discussed until consensus was reached. The following steps were taken for further data analysis:

1. Accelerometer recordings of ET patients 1–5, PD patients 1–5, and HC subjects 1–5 were used to evaluate the methods (Training set) and parameter settings.
2. Accelerometer recordings of ET 6–10, PD 6–10, and HC 6–10 are used to validate the selected method (Validation Group 1).
3. EMG recordings of all subjects were used to validate the selected method (Validation Group 2).

### 2.4. Tremor Classification

The tremor classification method split the data into TW and NTW, based on the power distribution within the tremor frequency band. For each 3-s window, the PSD was estimated for the frequency range 3.5–12 Hz. Then, the relative power of the tremor frequency $P_{tremor,rel}$ was calculated according to (1):

(1) $$P_{tremor,rel}=\frac{P\left(f_{tremor}\right)}{P\left(3.5-12\;\mathrm{Hz}\right)}.$$

With $f_{tremor}$, the frequency range from $f_{peak}-0.5\;\mathrm{Hz}$ and $f_{peak}+0.5\;\mathrm{Hz}$. $f_{peak}$ was the detected peak within the tremor frequency range 3.5–12 Hz. A window was classified as a TW when the relative power within a 1 Hz range around the peak frequency exceeded or was equal to the thresholds defined in Table 2.

$$\mathrm{TW}\;\;P_{tremor,rel}\geq Threshold_{RelP},$$

$$\mathrm{NTW}\;\;P_{tremor,rel}<Threshold_{RelP}.$$

In case an even power distribution existed within the tremor frequency band, the relative power around the peak frequency was approximately 0.11. The thresholds to be tested were set to: 0.35, 0.40, 0.45, and 0.50 to ensure that the power at the peak was at least three times higher compared to the rest of the frequency band (3.5–12 Hz).

### 2.5. Outcome Parameters

The sensitivity, specificity, and accuracy achieved with both methods were calculated with respect to this visual classification using a confusion matrix. For each task and hand, 36 windows were classified, resulting in a total of 3240 windows for the Training set and the same amount for Validation Group 1. For Validation Group 2, 6480 windows were available.

(2) $$\mathrm{Sensitivity}=\frac{\mathrm{TP}}{\mathrm{TP}+\mathrm{FP}},$$

(3) $$\mathrm{Specificity}=\frac{\mathrm{TN}}{\mathrm{TN}+\mathrm{FN}},$$

(4) $$\mathrm{Accuracy}=\frac{\mathrm{TP}+\mathrm{TN}}{\mathrm{TP}+\mathrm{FP}+\mathrm{TN}+\mathrm{FN}}.$$

With TP, the correctly identified TW, FP, the windows falsely identified as TW, TN, and the correctly identified NTW and FN the windows falsely identified as NTW. For the EMG data, 6480 windows were classified as either TW or NTW using the most suitable method determined with the accelerometer data. Furthermore, the following three parameters were determined for TW and NTW: TSI, tremor power, and tremor frequency.

Compared to di Biase et al. [14], the method to calculate the TSI was slightly adapted. Instead of calculating the instantaneous frequency from the time signal, we calculated the peak frequency between 3.5–12 Hz (*periodogram* routine in MATLAB 2013a) using a sliding window of 1 s over each 3-s window with 75% overlap. The large overlap was chosen to approximate the instantaneous frequency used by di Biase et al. [14]. The TSI was calculated as the difference between $f_{peak}$ of two successive windows. 

### 2.6. Statistical Analysis

To compare the TSI between groups during TW and NTW, the Kruskal-Wallis test was used. 

## 3. Results

A paired-sample t-test reveals no significant differences in age between groups (p < 0.05; PD – ET: p = 0.54; PD – HC: p = 0.22; ET – HC: p = 0.42). Gender is not considered to have any influence on the results.

Figure 1 shows ten s of the accelerometer signal (left) and the PSD of the 3-s windows of the whole signal (right). Data of a random tremor patient (grey) and a typical HC (black) are displayed. 

From top to bottom, data recorded during the RT, PT, and MT are visible. During the RT and PT, the amplitude and amplitude fluctuations are larger in the tremor patient compared to the HC. The power is several times smaller in the HC subject compared to the tremor patient. Therefore, an enlargement of the PSD of the HC subject in the tremor frequency band is given in the right column. During all three tasks, a clear peak around 5 Hz (and higher harmonics) is seen in the tremor patient but not in the HC. During the MT, a peak is visible in the lower frequency range in both groups, corresponding to the voluntary movement made by the subjects. In the tremor patient, additional peaks around 5 and 10 Hz are seen.

### 3.1. Sensitivity, Specificity, and Accuracy

In Table 2, the results of the method Trainings and validation are given. In the Training set, the highest accuracy (all three tasks combined) is achieved with a threshold of 0.40 for the tremor classification method, accuracy was 90.80% (Table 2–mid column). Overall, the highest accuracy was achieved in Validation Group 2, at 94.38% (Table 2–right column). 

### 3.2. The Tremor Stability Index

**Figure 2:**
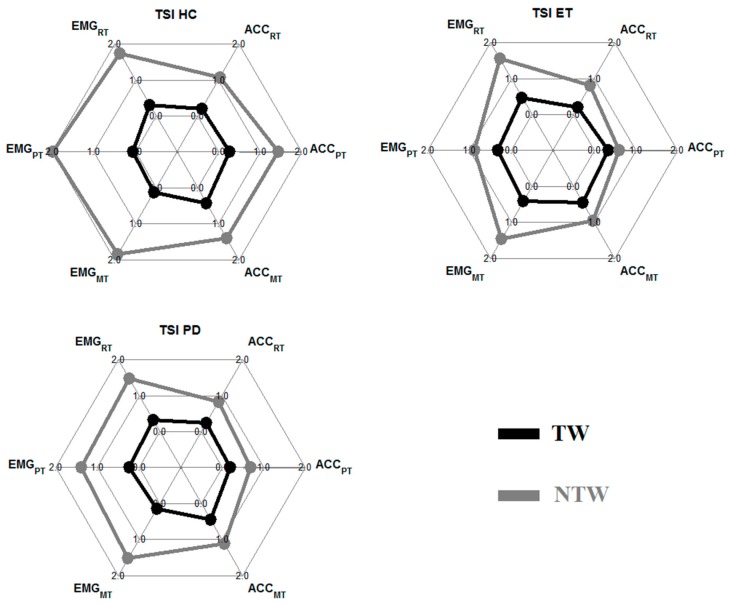
Group mean tremor stability index (TSI) of the healthy control (HC), essential tremor (ET), and Parkinson’s disease (PD) groups. In each spider plot on the left side are the results of the EMG data (top to bottom: RT, PT, and MT), and on the right side are the results of the accelerometer data. In **black**, the TW results are displayed, and in **grey**, the results of the NTW.

In Figure 2, the results of the TSI of each group (HC: left; ET: mid; PD: right) during tremor (black) and non-tremor (grey) windows are given. Results are displayed for the EMG (left side spider plot) and the ACC (right side spider plot) during all tasks.

The TSI was significantly different between the HC group and the two patient groups during the PT for TW (EMG) and NTW (ACC). An overview of all significant p-values (p < 0.05) is given in Table 3.

## 4. Discussion

A transparent quantitative tremor detection method that reliably detects tremor in accelerometer and EMG data recorded under different movement conditions using a straightforward experimental setup was introduced. In literature, several methods have been used [11,15,22,23,24,25], but no objective golden standard has been determined yet. We showed that the same method can be used for several types of tremors during different tasks and for both EMG and accelerometer data. Second, we showed that splitting data into TW and NTW is desirable when determining parameters, such as the TSI.

### 4.1. Tremor Classification Method

The presented tremor classification method depends on the power distribution within the tremor frequency band. It was found that the higher the chosen threshold, the higher the specificity and the lower the sensitivity. But with a threshold of 0.40, both remain sufficiently high to classify TW and NTW. Some windows in the HC group are classified as TW (approximately 6.34%). This is in accordance with a study of Elble [26], who found that up to 8% of young and elderly adults have an EMG-acceleration pattern which is indistinguishable from an essential tremor. Windows with a clear peak in the tremor frequency band were classified as TWs. The tremor classification method was able to classify 78.31–92.12% of all TWs correctly, even those with a small amplitude. Low power physiological tremors were detected in HC but can be distinguished from pathological tremors based on their power distribution. The advantage of this method over others is that is can be used for EMG and accelerometers and has been tested in three different groups and during three different tasks. Misclassification is most often due to a second peak in the frequency band. Two reasons for a second peak are a slight shift in tremor frequency during the 3 s and the presence of higher harmonics of the tremor frequency. If these peaks have a high power, classification is difficult. Overall, small and large amplitude tremors could be well detected. Due to its robustness, the method could also be tested for ambulatory recording of tremors in a future study. 

### 4.2. Tremor Measures

For TWs and NTWs, the TSI were calculated (see Figure 2). The goal was to prove the advantage of splitting data into TWs and NTWs. Analysis of NTW showed that there are almost no significant differences between the groups. Only during the PT was the TSI in the ET group significantly smaller compared to the HC group (EMG data), and the TSI was significantly smaller in both patient groups compared to the HC group for the accelerometer data. During the MT, ET subjects also had a significantly smaller TSI during NTW compared to the HC group. The tremor frequency during the RT in NTW was significantly higher in the patient groups compared to the HC group. In the HC group, the mean frequency was around 3.5 Hz. This was probably caused by decreasing power with increasing frequency (3.5 Hz being the lowest frequency in the tremor band) and not by actual tremors.

During detected TWs, the only significantly different results found were for the EMG data during the PT and MT. This is in contrast to the good results of di Biase et al. [14], who were able to distinguish between ET and PD using receiver operating characteristic analysis (area under the curve: 0.916). Differences might be the result of the definition of TWs and the length of the recording. Di Biase et al. [14] used 100 s of tremor recordings, and we used 60 s in total, splitting those 60 s into TWs and NTWs. This resulted in a total of 2262 (ACC) and 1529 (EMG) TW over all subjects. Another cause of the discrepancy might be that di Biase et al. [14] used the instantaneous frequency determined from the time signal instead of the frequency of a 3-s window determined from the periodogram. However, the results also show that the differences between groups in TSI is a lot larger in the TW compared to the NTW. This suggests that without splitting of the data prior to determining tremor parameters, in this case the TSI, the differences between groups would be even smaller. Furthermore, the results showed that the value of the TSI, and probably other tremor parameters as well, is sensor-type and task-dependent. 

## 5. Conclusion

Overall the method used is suitable to split data into TWs and NTWs using EMG and accelerometer data streams. Splitting data into TWs and NTWs proved useful for the analysis of the TSI. During TWs, the differences in parameters between groups was larger compared to NTWs. Furthermore, the postural task is the most useful task to differentiate between groups. For future analysis longer data sets should be used to be able to compare the results of the TSI better to the results of di Base et al. [14]. However, this study also shows the importance of reliable tremor detection for parameters, such as the TSI. Especially in the early stages of the disorders, tremor detection can be challenging and classification of TWs difficult. The method used here proved reliable in detecting even small amplitude pathological tremors. 

## Figures and Tables

**Table 1:** Overview of patients’ details.

Subject	Gender	Age	Disease Onset	Medication
* PD 1	M	58	50	Levodopa, Trihexyphenidyl
* PD 2	M	69	64	Rasagiline, propranolol
* PD 3	M	67	63	Trihexyphenidyl
* PD 4	F	81	76	Levodopa-Carbidopa, metoprolol tartrate
* PD 5	F	62	60	Levodopa
Ϯ PD 6	M	49	47	Levodopa-Carbidopa, ropinirole hydrochloride
Ϯ PD 7	M	71	71	-
Ϯ PD 8	F	43	40	Trihexyphenidyl, ropinirole hydrochloride
Ϯ PD 9	M	78	76	Levodopa-Carbidopa, Rasagiline, perindopril, omeprazole, pravastatin
Ϯ PD 10	M	68	60	Levodopa-Carbidopa
* ET 1	M	45	Childhood	-
* ET 2	F	81	Childhood	-
* ET 3	M	85	Childhood	Propranolol
* ET 4	M	65	Teenager	-
* ET 5	F	51	Childhood	-
Ϯ ET 6	M	49	40	Propranolol
Ϯ ET 7	M	54	Teenager	-
Ϯ ET 8	M	70	Childhood	-
Ϯ ET 9	M	64	Teenager	-
Ϯ ET 10	M	55	Teenager	-

(*) Training set. (Ϯ) Validation group. M = Male; F = Female.

**Table 2:** Tremor classification method—training and validation.

	Threshold (Training Set)				ValGroup 1	ValGroup 2
	0.35	0.40	0.45	0.50	0.40	0.40
Sensitivity (%)	92.64	84.84	76.09	66.20	78.31	92.12
Specificity (%)	87.13	96.45	99.10	99.70	95.00	95.00
Accuracy (%)	89.81	90.80	87.90	83.40	90.06	94.38
TW HC (%)	7.22	1.20	0	0	6.76	1.62

Sensitivity, specificity, accuracy and tremor windows (TWs) detected in the Training set (middle column) for all threshold settings. The right column contains the results of the validation groups: accelerometer data of Validation Group 1 (marked with Ϯ in Table 1) and EMG data of all subjects (Validation Group 2). HC = healthy controls; TW = Tremor window; ValGroup 1 = Validation Group 1; ValGroup 2 = Validation Group 2.

**Table 3:** Results of the statistical analysis.

Parameter	Task	p-Value TW		p-Value NTW	
		EMG	ACC	EMG	ACC
TSI	RT	-	-	-	-
	PT	< 0.001 ^Ϯ^	-	< 0.001 *	< 0.001 ^Ϯ^
	MT	0.004 *	-	-	0.02 *

An overview of the results of the statistical analysis of the TSI. Only statistically significant results are given (p < 0.05). The asterisk (*) marks the results where the HC group was significantly different from the ET group. The (Ϯ) denotes the tasks/parameters where the HC group is significantly different from both patient groups.
